# An Aptamer-Based Antagonist against the Receptor for Advanced Glycation End-Products (RAGE) Blocks Development of Colorectal Cancer

**DOI:** 10.1155/2021/9958051

**Published:** 2021-05-05

**Authors:** Jihui Zheng, Wenjing Zhu, Fang He, Zhu Li, Na Cai, Hong-Hui Wang

**Affiliations:** ^1^College of Biology, Hunan University, Changsha, China; ^2^CellWay Bio, Changsha, China

## Abstract

Tumor angiogenesis plays a crucial role in colorectal cancer development. Dysregulation of the receptor for the advanced glycation end-products (RAGE) transmembrane signaling mediates inflammation, resulting in various cancers. However, the mechanism of the RAGE signaling pathway in modulating development of colorectal cancer has not been explored. In this study, an aptamer-based RAGE antagonist (Apt-RAGE) was used to inhibit interaction between RAGE and S100B, thus blocking downstream NF*κ*B-mediated signal transduction. *In vitro* results showed that Apt-RAGE effectively inhibited S100B-dependent and S100B-independent RAGE/NF*κ*B activation in colorectal HCT116 cancer cells, thus decreasing proliferation and migration of cells. Notably, expression and secretion of VEGF-A were inhibited, implying that Apt-RAGE can be used as an antiangiogenesis agent in tumor therapy. Moreover, Apt-RAGE inhibited tumor growth and microvasculature formation in colorectal tumor-bearing mice. Inhibition of angiogenesis by Apt-RAGE was positively correlated with suppression of the RAGE/NF*κ*B/VEGF-A signaling. The findings of this study show that Apt-RAGE antagonist is a potential therapeutic agent for treatment of colorectal cancer.

## 1. Introduction

Colorectal cancer is a multifactorial disease that affects over four million people worldwide [1]. Previous studies report that the development of colorectal cancer is highly correlated with tumor angiogenesis [2] which is implicated in the pathological process of many critical diseases [3]. Inhibition of angiogenesis is associated with alleviation of cancer progression and metastasis [4, 5]. New blood vessels formed during angiogenesis may contribute to inflammation-associated carcinogenesis, inducing tumor progression and tumor metastasis [6, 7]. Unregulated release of different inflammatory mediators in the tumor microenvironment (TME) activates growth, proliferation, and migration of colorectal cancer cells [8]. In addition, inflammation mediators in TME induce abnormal angiogenesis by increasing secretion of vascular endothelial growth factor (VEGF). Moreover, the receptor for advanced glycation end-products (RAGE) is positively correlated with increased vessel density and progression of colorectal cancer, implying that the RAGE signaling pathway participates in VEGF-mediated angiogenesis [9].

RAGE is a type I transmembrane receptor present in diverse cell types and functions as a pattern-recognition receptor [10]. Interaction of RAGE with its various ligands (including AGE, HMGB1, and S100B) promotes cancer cell growth, invasion, and angiogenesis [3]. The S100 protein ligand activates the RAGE signaling thus promoting survival and proliferation of cancer cells [11]. In addition, overexpression of RAGE promotes colon cancer malignancy by accelerating proliferation and migration of cancer cells through activation of nuclear factor-kappa B- (NF*κ*B-) mediated transcription leading to inflammation-associated carcinogenesis [12–14]. Furthermore, S100B plays an essential role in colon carcinogenesis by promoting NF*κ*B-mediated transcription through the RAGE signaling, affecting various phenotypes of cancer such as proliferation, metastasis, and angiogenesis [15]. Therefore, studies should explore novel specific therapeutic agents targeting S100B/RAGE/NF*κ*B axis to block development of colorectal cancer.

Cell surface receptor targeting strategies have explored aptamers as novel candidates for targeted cancer therapy. Aptamers have various advantages over protein-based drugs, such as antibodies or peptides [16]. Aptamers are short and single-stranded oligonucleotides and have been reported to be antagonists against various target proteins through phylogenetic and exponential enrichment (SELEX) analysis [17]. Aptamers are chemical antibodies with three-dimensional structures; therefore, they have excellent thermal stability and high affinity and specificity for the homologous protein targets and display good biocompatibility [18, 19]. Therefore, in this study, we developed an aptamer-based strategy for use as a molecular inhibitor of RAGE/NF*κ*B/VEGF-A axis for suppression of inflammatory-induced angiogenesis associated with colorectal cancer progression.

The aim of this study was to design an aptamer against RAGE as a new antagonist to selectively inhibit RAGE/NF*κ*B signaling transductions. Analysis showed that the aptamer-based antagonist inhibited proliferation and migration of colorectal cancer cells induced by S100B. In addition, it decreased synthesis and secretion of VEGF-A protein, which is implicated in tumor angiogenesis. The *in vivo* results indicated that the aptamer of RAGE exhibited excellent inhibition activity on development of colorectal tumors, by suppressing angiogenesis and microvasculature formation in xenograft nude mice. The findings of this study, therefore, show that the novel aptamer against RAGE is a potential therapeutic agent for treatment of colorectal cancer.

## 2. Material and Methods

### 2.1. Reagents

The fetal bovine serum (FBS) was purchased from Biological Co., Ltd.; penicillin-streptomycin (100x), 0.25% trypsin-EDTA (1x), serum-free cryopreservation fluid, 3-(4,5-dimethyl-2-thiazolyl)-2,5-diphenyl-2-H-tetrazolium bromide (CCK-8), ECL luminescent agents, color pre-dyed protein marker, and antibody diluent were purchased from New Cell & Molecular Biotech Co., Ltd.; recombinant protein S100B (*E. coli*, N-6His) was purchased from Novoprotein Scientific Inc. RIPA lysis buffer was purchased from Beyotime Biotechnology. Phosphatase inhibitors and protease inhibitors were from Bimake; nitrocellulose membrane was purchased from Merck Millipore Company; antibodies against phosphorylated NF*κ*B p65 (Ser 536), phosphorylated Akt (Ser 473), and phosphorylated ERK1/2 (Thr202/Tyr204) were purchased from Cell Signaling Technology (Beverly, Massachusetts, USA). The polyclonal antibodies for VEGF-A, RAGE, and CD31 were from Santa Cruz Biotechnology (Santa Cruz). The primary monoclonal antibody against *α*-tubulin was purchased from CellWay technology (CellWay Bio). The secondary antibodies including HRP-conjugated goat anti-mouse IgG (H&L) and HRP-conjugated goat anti-rabbit IgG (H&L) were from Invitrogen. PrimeScript™ RT reagent kit (perfect real time), Takara RR037A kit, EasyGeno rapid recombinant clone kit, endotoxin-free plasmid extraction kit, and DNA gel recovery kit were obtained from Tiangen.

### 2.2. Cell Culture

HCT116 cell, a colon cancer cell line, was cultured in Dulbecco's modified Eagle's medium (DMEM) with 10% FBS and 1% penicillin/streptomycin. All cells were incubated in a humidified atmosphere at 37°C with 5% CO_2_.

### 2.3. Preparation of Apt-RAGE

The aptamer against RAGE (Apt-RAGE) was adopted based on the systematic evolution of ligands by exponential enrichment (SELEX) previously. [20] Sequences of Apt-RAGE and the control DNA aptamer (Ctrl-aptamer) are as follows. Apt-RAGE: 5′-CCTGATATGGTGTCACCGCCGCCTTAGTATTGGTGTCTAC-3′ and Ctrl-aptamer: 5′-TTCGGCCTGGGGGCGGCCAGTTCGGGTCCAGTCGCGGGAG-3′.

### 2.4. Molecular Cloning

The cloning primers (Supplemental Table 1) were designed according to the CDS sequence of the gene AGER (Homo sapiens gene of *rage*). The cDNA fragment of the RAGE was amplified by PCR and cloned into the plasmid of pcDNA 3.1 vector. The plasmids of pcDNA 3.1 RAGE were isolated and identified by Sanger sequencing (Tsingke Biotech Co., Ltd.).

### 2.5. Plasmid Transfection

Cells were inoculated and transfected into a 6-well plate culture dish (Wuxi NEST Biotechnology Co., Ltd.) with about 2 × 10^5^ cells per well on the day before transfection, and the cell density could reach about 70-80% on the next day. The mixture of Lipo8000™ Transfection reagent and plasmids was dripped into the cells in the six-well plate. After 6 hours, the culture medium was changed for 48 h culturing.

### 2.6. Western Blotting

Cells were seeded in a 6-well plate culture dish (NEST Biotech Co., Ltd.). Before treatment, the cells were starved for 24 h by incubation with the DMEM containing 0.2% FBS. Subsequently, the cells were pretreated with Ctrl-aptamer or Apt-RAGE for one hour, then treated with S100B for 30 min in the incubator, and then lysed with RIPA lysis buffer. The cell lysates were centrifuged at 14000 rcf for 10 min at 4°C and separated by 10% SDS-PAGE electrophoresis followed by transferring to a nitrocellulose membrane by semidry electrophoretic transfer unit. Each membrane was blocked with 5% skim milk in PBST (1× PBS with 0.1% Tween-20) at room temperature for 1.5 h and incubated with different primary antibodies (1 : 1000 dilution) overnight at 4°C. Subsequently, the membranes were incubated with horseradish peroxidase-conjugated secondary antibodies at room temperature for 1 h. Finally, the membranes were reacted with ECL substrate solution (NCM Biotech Co., Ltd.) and the chemiluminescent images were acquired and analyzed using Bio-Imaging Systems (MicroChemi4.2).

### 2.7. Cell Viability Assay

The CCK8 assay was carried out to determine relative cell viability. [21] HCT116 cells were seeded at 5 × 10^3^ cells/well on a 96-well plate culture dish (Wuxi NEST Biotechnology Co., Ltd.) and allowed to adhere for 24 h at 37°C in 5% CO_2_. The cells were pretreated with Apt-RAGE (100 nM) and then treated with S100B (2 ugs/mL), S100B within the fresh medium for 24 h, 48 h, or 72 h. After the incubation, cells within the 96-well plate were incubated with CCK8 solution for 1-3 h at 37°C in 5% CO_2_, followed by measurement using a GENios Microplate Reader (TECAN) at the absorbance at 450 nm.

### 2.8. Wound Healing Assay

3 × 10^4^ HCT116 cells were seeded into a 12-well plate culture dish (Wuxi NEST Biotechnology Co., Ltd.) for 90% confluence and scratched using a 200 *μ*L tip. After scratching, the cells were washed with PBS twice, and then the medium containing Apt-RAGE (100 nM) was added. After 1 h, S100B was added to the culture medium. Under an inverted microscope, the scratched cells were photographed after 0 h, 24 h, and 48 h. The wound closure rate was calculated as follows: Migration rate (%) = (Scratch distance at 0 h − Scratch distance at indicated time)/Scratch distance at 0 h × 100.

### 2.9. Transwell Assay

The migration ability of HCT116 cells was assessed by using Transwell chamber (BD Biosciences, San Diego, CA). 500 *μ*L of complete medium containing 10% FBS was supplemented into the lower chamber (the bottom of a 24-well plate), then the medium containing Apt-RAGE (100 nM) or Ctrl-aptamer. Next, 5 × 10^4^ cells/well were added into the upper chamber. S100B was added into the lower chamber. After 24 h incubation, the migrated cells were stained with crystal violet. Five random fields at 200x magnification were used for cell counting for each membrane.

### 2.10. Measurement of VEGF-A by ELISA

ELISA was performed to determine VEGF-A production according to the user's manual of a Human VEGF-A ELISA kit (ABclonal Technology).

### 2.11. Xenograft Studies

The animal experiments were approved by the ethical committee of College of Biology, Hunan University, China, and performed according to the *Guide for the Care and Use of Laboratory Animals* of the National Institutes of Health. The HCT116 cells (2 × 10^6^ cells) were intradermally injected into the upper flank of female 6-week nude mice (*n* = 20). 2 days posttumor inoculation, Apt-RAGE (38.4 pmol/day/g body weight, *n* = 5) or vehicle (*n* = 5) was injected adjacent to the tumor daily for 12 days. The volume of tumors and body weight were measured daily. The tumor volume (mm^3^) = [(width)/2 × length/2] mm^3^. At 12 days posttumor inoculation, mice were humanely sacrificed by isoflurane inhalation, and the HCT116 tumor section was excised for immunohistochemical staining.

### 2.12. Immunohistochemical Staining

Harvested tumors and paracancerous tissue were embedded in the optimal cutting temperature compound (OCT, Tissue-Tek, Sakura), stored at −80°C. Immunohistochemistry was carried out using a two-step ELISA Kit (mouse/rabbit-enhanced polymer system) (ZSGB-BIO). Primary antibodies include RAGE (1 : 50 dilution), VEGF-A (1 : 50 dilution), p-NF*κ*B (1 : 50 dilution), and CD31 (1 : 50 dilution). The specimens were stained using the DAB kit (ZSGB-BIO) and then imaged using a digital slicing system (Pannoramic MIDI).

### 2.13. Statistical Analysis

All values were presented as the means ± SEM. Statistical significance was evaluated using the Student *t*-test for paired comparison (GraphPad Prism 6); ^∗^*p* < 0.05 and ^∗∗^*p* < 0.01 were considered to be significant.

## 3. Results

### 3.1. RAGE Expression Correlates with Microvasculature Formation in Colorectal Cancer

**Figure 1:**
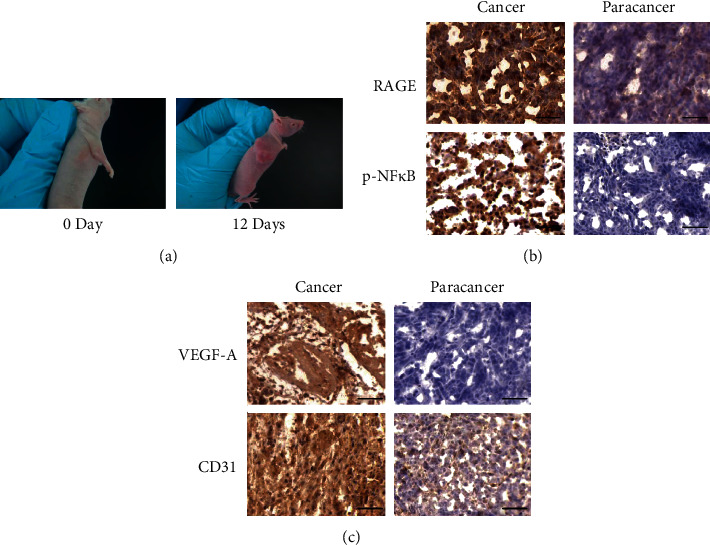
Association of RAGE expression with tumor angiogenesis in colorectal cancer development. (a) HCT116 cells were intradermally injected into the upper flank of 6-week female nude mice to establish colorectal cancer xenograft model. (b) Immunohistochemical analysis of cancer tissues and the paracancer tissues from nude mice using antibodies against RAGE and p-NF*κ*B. Scale bar: 50 *μ*m. (c) Immunohistochemical analysis of cancer tissues and the paracancer tissues from nude mice using antibodies against VEGF-A and CD31. Scale bar: 50 *μ*m.

Tumor-associated angiogenesis is associated with tumor growth and development *in vivo* [22]. A colorectal tumor-bearing nude mouse model was established to explore the role of RAGE in tumor-associated angiogenesis (Figure 1(a)). Expression of RAGE and phosphorylation of NF*κ*B were analyzed through immunohistochemistry staining of tumor specimens prepared post 12 days after tumor inoculation days. Analysis of staining results showed increase in expression level of RAGE protein and significant phosphorylation of NF*κ*B in colorectal tumor tissue compared with those in paracancer normal tissue (Figure 1(b)). Notably, CD31-positive blood vessels formed adjacent to tumor tissues, and a significant increase in the level of VEGF-A was observed compared with those in the adjacent normal tissue (Figure 1(c)). These findings imply that development of colorectal cancer may be mediated by RAGE/NF*κ*B/VEGF-A axis, which plays a role in promoting tumor angiogenesis.

### 3.2. Apt-RAGE Blocks the NF*κ*B Signaling Pathway and VEGF-A Secretion

**Figure 2:**
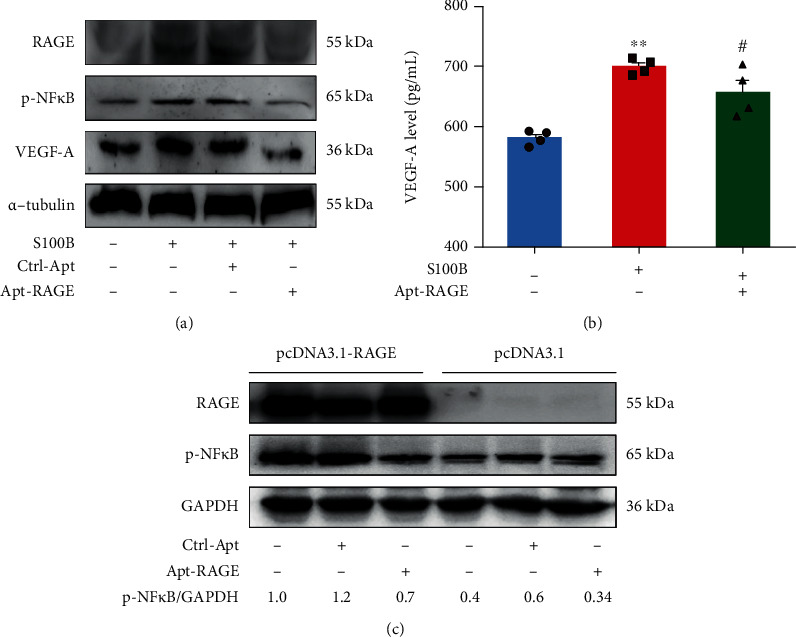
Inhibitory effect of Apt-RAGE on S100B-induced synthesis and secretion of VEGF-A protein. (a) HCT116 cells were starved and pretreated with 100 nM Apt-RAGE or Ctrl-Apt for 90 min in the presence of S100B (2 *μ*g/mL). Phosphorylation of NF*κ*B, VEGF-A, and RAGE was examined using western blotting. (b) ELISA assay was used to determine VEGF-A release in the culture medium treated with S100B (2 *μ*g/mL) in the presence or absence of Apt-RAGE (100 nM) or Ctrl-Apt (100 nM). Data are presented as the means ± SEM, ^∗∗^*p* < 0.01 vs. untreated control and ^#^*p* < 0.05 in Apt-RAGE vs. S100B. (c) Apt-RAGE inhibited S100B-independent phosphorylation of NF*κ*B in RAGE-overexpressed cells. HCT116 cells transfected with pcDNA3.1-RAGE or pcDNA3.1 for 48 h and incubation with Apt-RAGE (100 nM) for 20 min. Phosphorylation of NF*κ*B and the expression of VEGF-A and RAGE were examined using western blotting.

The role of RAGE signaling in tumorigenesis was explored using *in vitro* cultured colorectal cells. S100B, a ligand of RAGE and a known mediator of inflammation, significantly induced phosphorylation of NF*κ*B and expression of VEGF-A (Figure 2(a)). It has been previously reported that the RAGE-NF*κ*B signaling plays an essential role in VEGF-A secretion [23]. In this study, we explored the relationship between S100B and VEGF-A secretion using HCT116 cells. ELISA analysis showed that S100B enhanced VEGF-A secretion in the culture medium (Figure 2(d)), implying that VEGF-A secretion is mediated by induction of the RAGE/NF*κ*B signaling pathway by S100B. We proposed that the aptamer-based RAGE antagonist (Apt-RAGE) may inhibit the interaction between RAGE and S100B to block downstream NF*κ*B-mediated signal transduction. The aptamer against RAGE was previously screened and was used to block the AGE-RAGE signaling, which efficiently attenuated the development experimental diabetic nephropathy [20]. We characterized the stability of Apt-RAGE in 10% serum and found that the Apt-RAGE remained stable without degradation in 5 hours, which could be enough for cell experiment and in vivo study (Figure S1). Based on the Mfold software simulation, two predicted secondary structures were generated by free energy minimization using the RNA folding algorithm (Figure S2). The calculated free energy for each aptamer is ΔGA = −2.07 kcal/mol and ΔGB = −1.39 kcal/mol. Notably, pretreatment of HCT116 cells with 100 nM Apt-RAGE significantly inhibited S100B-induced phosphorylation of NF*κ*B and increased in VEGF-A protein level compared with the Ctrl-Apt-treated group (Figure 2(a)). We also examined other signaling pathways and found that Apt-RAGE failed to affect the Akt and ERK signaling, suggesting the specificity of the Apt-RAGE to block the RAGE/NF*κ*B signaling (Figure S3). Furthermore, we confirmed that Apt-RAGE suppressed secretion of VEGF-A in culture medium (Figure 2(b)). These findings imply that Apt-RAGE inhibits VEGF-A release by inhibiting the RAGE/NF*κ*B signaling pathway.

A previous study reports that overexpression of RAGE affects downstream signaling thus promoting proliferation of hepatic cancer cells in a ligand-independent manner [24]. To explore the effect of high overexpression of RAGE on the NF*κ*B signaling in colorectal cancer cells, HCT116 cells were transfected with pcDNA-3.1-RAGE to overexpress human RAGE protein. Overexpression of RAGE significantly increased phosphorylation of NF*κ*B in pcDNA 3.1 RAGE-transfected cells compared with the control cells transfected with an empty vector (pcDNA 3.1) lacking RAGE (Figure 2(c)). Analysis showed that Apt-RAGE significantly inhibited phosphorylation of NF*κ*B in HCT116 cells with overexpressed RAGE (Figure 2(c)). This finding indicates that Apt-RAGE can be used as a potent antagonist for RAGE protein to inhibit the S100B-dependent and S100B-independent RAGE/NF*κ*B signaling.

### 3.3. Apt-RAGE Inhibits S100B-Induced Proliferation and Migration

**Figure 3:**
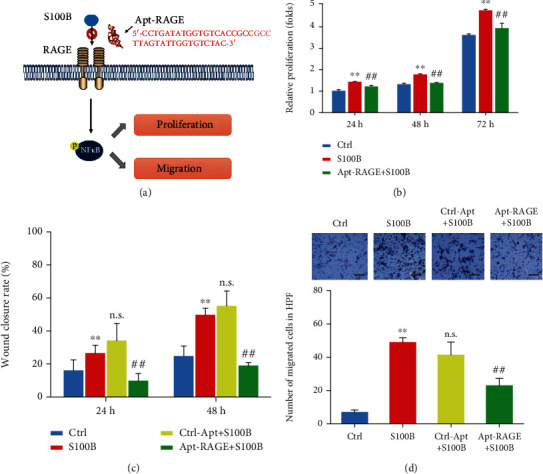
Apt-RAGE inhibited proliferation and migration of colorectal cancer cells. (a) Schematic illustration of Apt-RAGE effect on proliferation and migration of cancer cells. (b) Effect of Apt-RAGE (100 nM) on proliferation of HCT116 cells promoted by S100B (2 *μ*g/mL) was detected by CCK8 cell counting kit at 24 h, 48 h, and 72 h. Data are presented as the means ± SEM, ^∗^*p* < 0.01 vs. untreated control and ^#^*p* < 0.05 vs. S100B. (c) Quantitation of the effect of Apt-RAGE (100 nM) on migration induced by S100B (2 *μ*g/mL) in wound healing assay at 24 h or 48 h. ^∗^*p* < 0.01 vs. untreated group and ^##^*p* < 0.01 vs. S100B-treated group. n.s. indicates that the difference is not significant compared with the S100B-treated group. (d) Quantitative analysis of the effect of Apt-RAGE (100 nM) on directional migration induced by S100B (2 *μ*g/mL) evaluated using Transwell assay. Upper panel: representative image of the membrane with migrated cells, scale bar: 200 *μ*m. Lower panel: analysis of migrated cells using Transwell assay. Data are presented as the means ± SEM, ^∗∗^*p* < 0.01 vs. untreated control, ^##^*p* < 0.01 vs. S100B-treated group. n.s. indicates that the difference is not significant compared with the S100B-treated group.

Further, we explored the role of S100B on proliferation and migration of colorectal cancer cells (Figure 3(a)). Analysis showed that S100B significantly increased proliferation of HCT116 cells (2 *μ*g/mL) compared with that of controls (Figure 3(b)). Pretreatment with 100 nM Apt-RAGE significantly reduced proliferation of HCT116 cells (Figure 3(b)). To explore the effect of S100B on cell migration, a wound scratch assay was performed on a 2D interface. Analysis showed a significant increase in cell migration rate after treatment with S100B in a time-dependent manner compared with control cells (Figure 3(c), Figure S4). However, pretreatment with Apt-RAGE (100 nM) significantly reduced S100B-induced wound closure of HCT116 cells. Further, Transwell assay was performed to investigate the effect of Apt-RAGE on S100B-induced cell migration using a 3D interface. Analysis showed that Apt-RAGE significantly decreased the number of migrated cells compared with that of the S100B-treated group (Figure 3(d)). These findings show that Apt-RAGE effectively inhibits S100B-induced proliferation and migration of colorectal cancer cells.

### 3.4. Apt-RAGE Retards Development of Colorectal Cancer by Modulating Angiogenesis In Vivo

**Figure 4:**
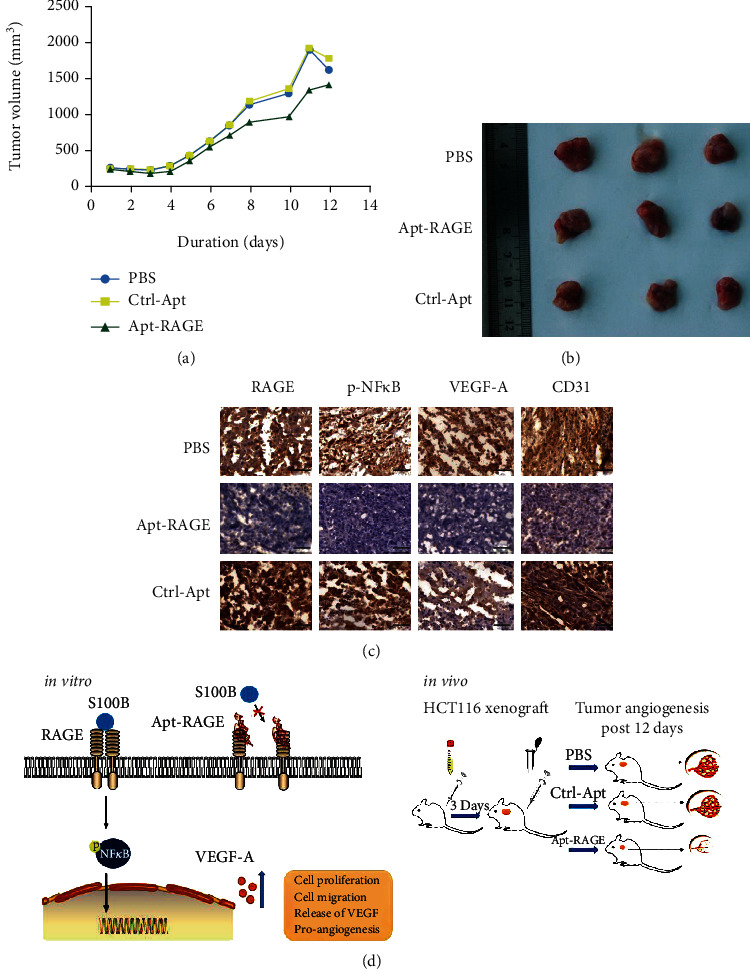
Effects of Apt-RAGE on angiogenesis of HCT116 cells xenograft nude mice. (a) The average volume of tumors treated with PBS (*n* = 5), Ctrl-Apt (*n* = 5), or Apt-RAGE (*n* = 5). Tumor volume was measured until the end of the experiments. (b) Images of representative tumors. (c) IHC staining was performed with RAGE, p-NF*κ*B, VEGF, or CD31 on the frozen sections. Scale bar: 50 *μ*m. (d) Schematic illustration of the role of Apt-RAGE to inhibit in vivo tumor angiogenesis (right) by blocking the S100B/RAGE/NF*κ*B/VEGF-A signaling pathway (left).

To investigate the effects of Apt-RAGE aptamer as an antagonistic agent *in vivo*, a colorectal tumor xenograft model was constructed. In summary, HCT116 cells were injected subcutaneously in mice to induce tumors. The tumor-bearing mice were administered with Apt-RAGE or Ctrl-aptamer daily for 12 days. The tumor volume from the mice treated with the Apt-RAGE was significantly smaller compared with that of the control group (Figure 4(a)). In the first four days, the tumor growth in the experimental group was similar to the control group, whereas growth gradually decreased from day five, compared with the PBS group and the Ctrl-aptamer group (Figure 4(b)). Analysis showed that Apt-RAGE significantly inhibited tumor growth. Furthermore, immunohistochemical analysis was performed showing that Apt-RAGE inhibited the RAGE level and phosphorylation of NF*κ*B, resulting in low VEGF-A levels compared with levels in the control group (Figure 4(c)). These findings indicate that Apt-RAGE inhibits tumor angiogenesis by blocking RAGE/NF*κ*B signal transduction.

## 4. Discussion

Development of colorectal cancer is associated with aberrant RAGE activation through angiogenesis-promoting TME inflammation. Therefore, RAGE is a potential therapeutic target for colorectal cancer treatment [9, 15]. RAGE binds to multiple ligands, including advanced glycation end-products (AGEs) and S100 proteins. After binding these ligands, it activates the downstream NF*κ*B pathway which is implicated in regulation of cell proliferation, survival, differentiation, and autophagy [25, 26]. In the present study, RAGE expression level was determined and the association with microvessel density in colorectal cancer tissue specimens was explored. The findings of this study showed that colorectal cancer tissues express high RAGE protein levels, which are positively correlated with increased microvessel density. In addition, phosphorylation NF*κ*B pattern was positively correlated with expression levels of VEGF and CD31 proteins in colorectal cancer tissue specimens.

S100B is used as a diagnostic marker for inflammatory malignant tumors. The S100B-induced signaling is positively correlated with development of various tumors [27, 28]. Moreover, VEGF-A promotes tumor angiogenesis during development of colorectal cancer [15]. This study explored the role of the S100B/RAGE signaling on cell viability, migration, and angiogenesis and progression of colorectal cancer. The findings of this study show that increased S100B protein levels are correlated with significant increase in proliferation and migration of human colon cancer cell *in vitro*. In addition, S100B activity significantly increased VEGF-A secretion from cultured cells. Apt-RAGE inhibited the RAGE signaling in colorectal cancer cells, thus inhibiting activation of NF*κ*B, tumor cell proliferation, migration, and release of VEGF-A. A previous study reported that RAGE is highly expressed in hepatoma cancer cells and is implicated in promoting proliferation of hepatoma cancer cells [24]. Similarly, the findings of this study showed that RAGE overexpression induces phosphorylation of NF*κ*B in the absence of S100B. Notably, analysis showed that Apt-RAGE inhibits S100B-independent NF*κ*B activation through inhibition of RAGE expression. Furthermore, the findings of this study indicate that Apt-RAGE inhibits S100B-RAGE-mediated angiogenesis by inhibiting S100B-induced activation of NF*κ*B. The inhibitory effect of Apt-RAGE on *in vitro* tumor angiogenesis was confirmed *in vivo* (Figure 4). *In vivo* experiments showed that Apt-RAGE inhibits phosphorylation of NF*κ*B and expression of VEGF, thus decreasing microvasculature which was analyzed through CD31-positive staining of the vascular endothelium in colorectal tumor specimens.

In conclusion, the findings of this study show that Apt-RAGE, an antagonist for RAGE, significantly inhibits synthesis and secretion of VEGF-A protein by inhibiting the NF*κ*B pathway in human colon cancer cells. Therefore, inhibition of Apt-RAGE on VEGF-A-mediated angiogenesis significantly decreases formation of microvasculature around tumors in xenograft model. In addition, Apt-RAGE inhibited S100B-dependent activation of proliferation and migration of colorectal cancer cells, which are critical events for cancer cells to adapt to the TME during tumor progression (Figure 4(d)). To the best of our knowledge, this is first study to report that Apt-RAGE inhibits proangiogenic and proliferative features of colorectal cancer cells. These results provide a basis for selective targeting of S100B/RAGE signaling using aptamer which is a novel approach to develop novel nucleic acid drugs for colon cancer therapy.

## Data Availability

The quantification data used to support the findings of this study are included within the article.

## Supplementary Materials

Supplementary Materials Supplemental Table 1: primer sequences for plasmid construction. Figure S1: the characterization of Apt-RAGE. (A) The molecular size of Apt-RAGE (Apt-RAGE) and Ctrl-aptamer (Ctrl-Apt). (B) The serum stability of Apt-RAGE. Figure S2: schematic representations of the secondary structures of the Apt-RAGE. Predicted secondary structures were generated by free energy minimization using the RNA folding algorithm Mfold (calculated free energy for each aptamer: ΔGA = −2.07 kcal/mol and ΔGB = −1.39 kcal/mol. Figure S3: effect of Apt-RAGE on the AKT and ERK signaling pathway in cultured HCT116 cells. Figure S4: effect of Apt-RAGE (100 nM) on the migration induced by S100B (2 *μ*g/mL) was detected by wound healing assay at 24 h or 48 h. Scale bar: 50 *μ*m.
