# Supplementary material for: An Aptamer-Based Antagonist against the Receptor for Advanced Glycation End-Products (RAGE) Blocks Development of Colorectal Cancer
Source: Mediators Inflamm. 2021 May 5;2021:9958051. doi: 10.1155/2021/9958051 (PMC8116144; doi:10.1155/2021/9958051)
Supplement: Supplementary Materials — Supplemental Table 1: primer sequences for plasmid construction. Figure S1: the characterization of Apt-RAGE. (A) The molecular size of Apt-RAGE (Apt-RAGE) and Ctrl-aptamer (Ctrl-Apt). (B) The serum stability of Apt-RAGE. Figure S2: schematic representations of the secondary structures of the Apt-RAGE. Predicted secondary structures were generated by free energy minimization using the RNA folding algorithm Mfold (calculated free energy for each aptamer: ΔGA = −2.07 kcal/mol and ΔGB = −1.39 kcal/mol. Figure S3: effect of Apt-RAGE on the AKT and ERK signaling pathway in cultured HCT116 cells. Figure S4: effect of Apt-RAGE (100 nM) on the migration induced by S100B (2 μg/mL) was detected by wound healing assay at 24 h or 48 h. Scale bar: 50 μm. [file 9958051.f1.pdf]

Supplemental Information

An aptamer-based antagonist against the receptor for advanced glycation end-products (RAGE) blocks development of colorectal cancer

Jihui Zheng[a], Wenjing Zhu[a], Fang He[a], Zhu Li [b], Na Cai[b*], Hong-Hui Wang[a*]

[a]College of Biology, Hunan University, Changsha, China

[b]CellWay Bio, Changsha, China

[*]Correspondence should be addressed to Na Cai; cainaya@hotmail.com and Hong-Hui Wang; wanghonghui@hnu.edu.cn

Supplemental Table 1 Primers sequences for plasmids construction

Primer name	Sequence（5'-3'）
RAGE-pcDNA3.1 forward	TGCTGGATATCTGCAGAATTCATGGCTGCCGGAACAGC
RAGE-pcDNA3.1 reverse	TTCGGGCCCAAGCTTGGTACCAGGCCCTCCAGTACTACTCT

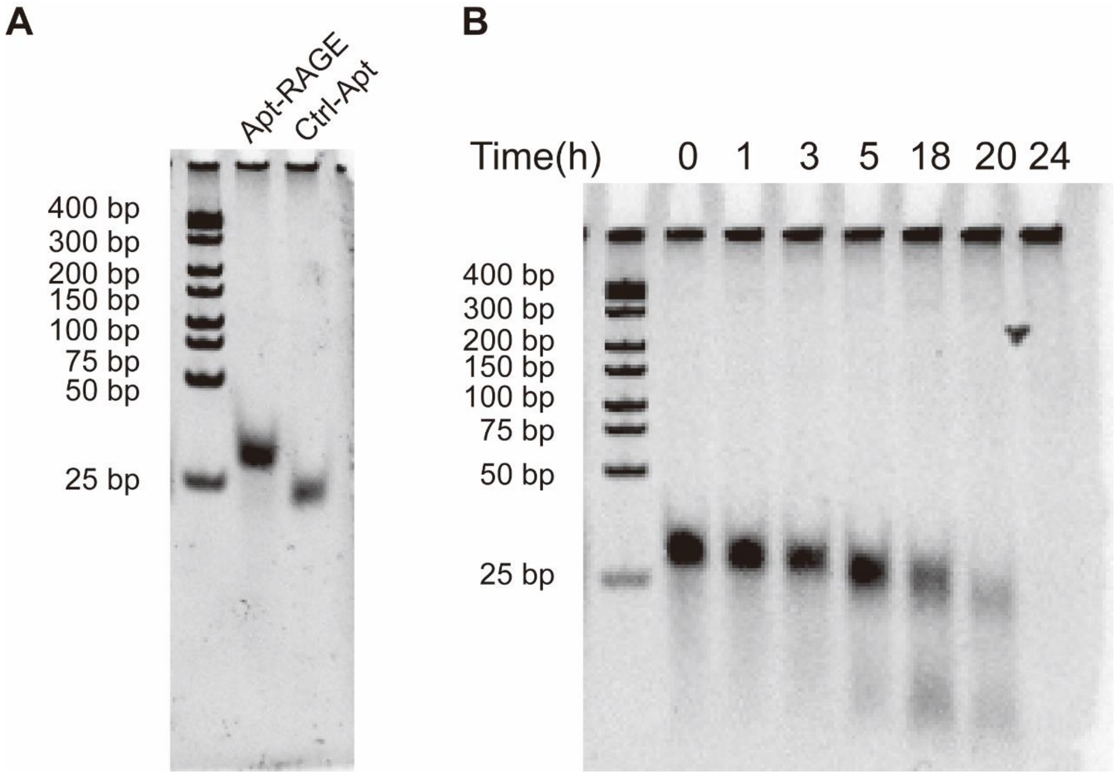

Fig. S1. The characterization of Apt-RAGE. (A) The molecular size of Apt-RAGE (Apt-RAGE) and Ctrl-Aptamer (Ctrl-Apt). (B) The serum stability of Apt-RAGE.

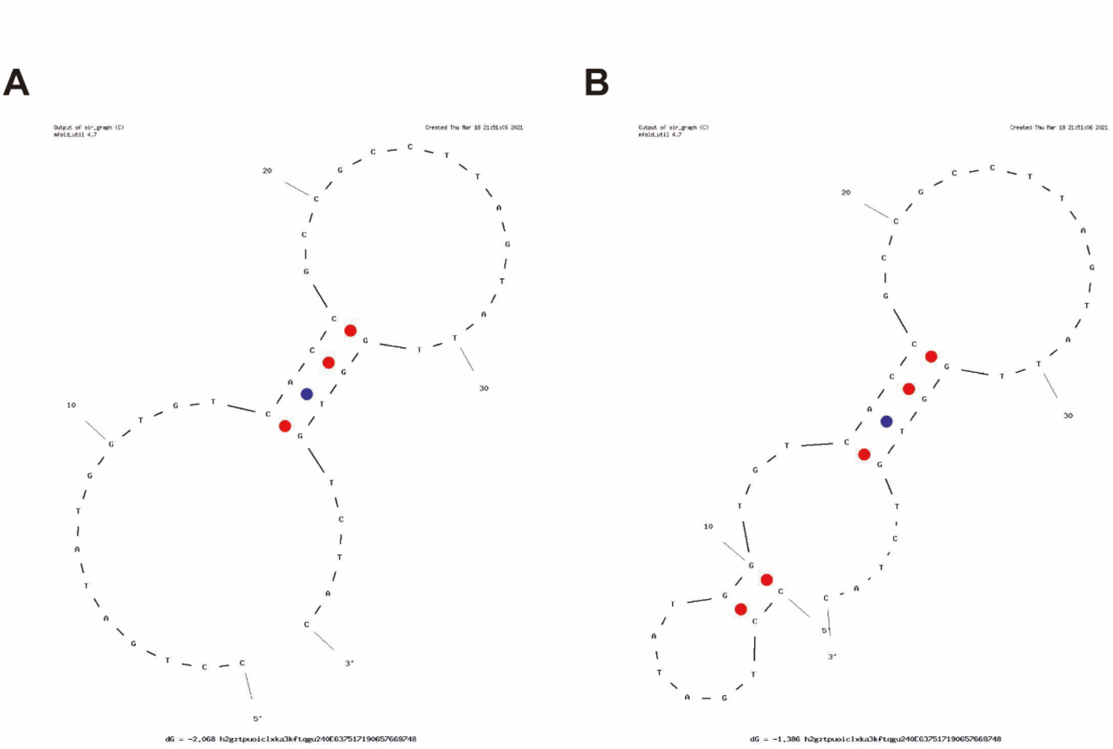

Fig. S2. Schematic representations of the secondary structures of the Apt-RAGE. Predicted secondary structures were generated by free energy minimization using the RNA folding algorithm Mfold (calculated free energy for each aptamer: ΔGA = -2.07 kcal/mol, ΔGB = -1.39 kcal/mol.

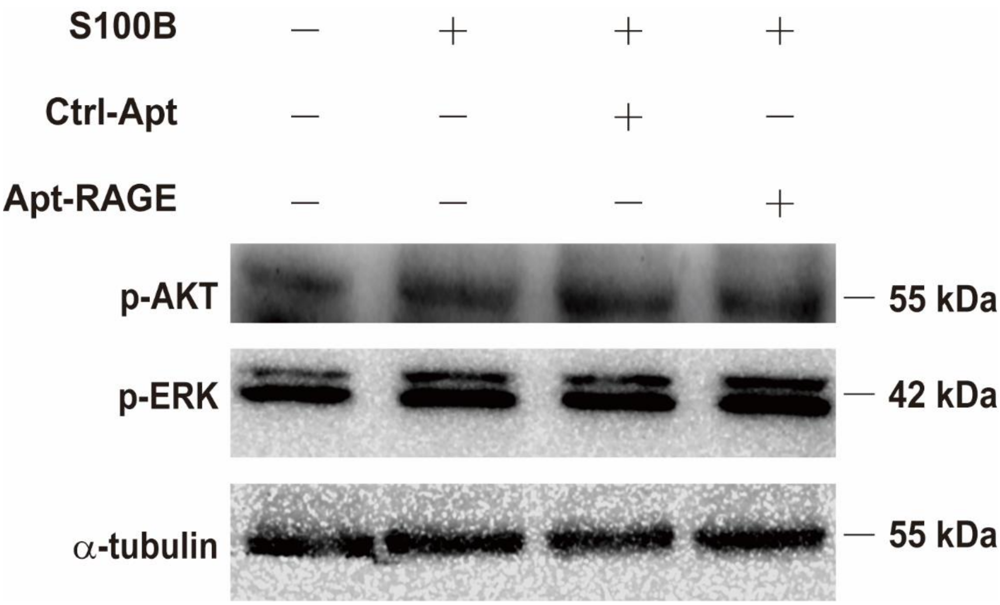

Fig. S3. Effect of Apt-RAGE on AKT and ERK signaling pathway in cultured HCT116 cells.

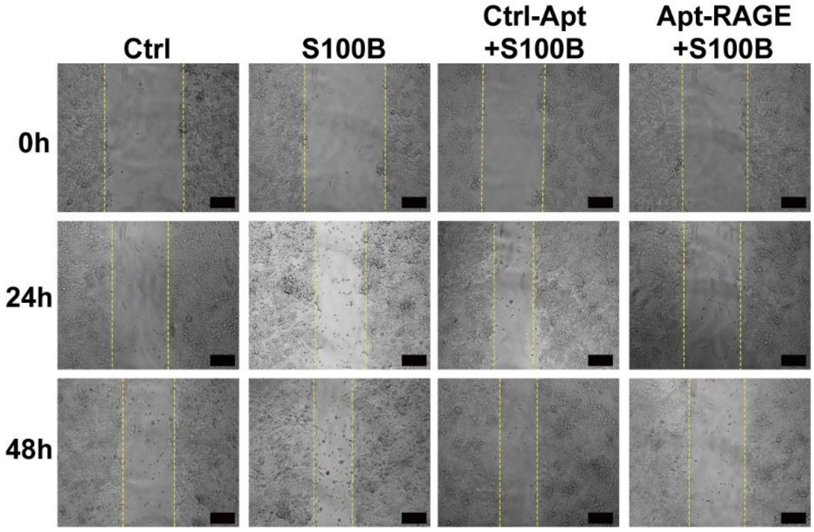

Fig. S4. Effect of Apt-RAGE (100 nM) on the migration induced by S100B (2 μg/mL) were detected by wound healing assay at 24 h or 48 h. Scale bar: 50 μm.
